# Too Dense or Not Too Dense: Higher Planting Density Reduces Cannabinoid Uniformity but Increases Yield/Area in Drug-Type Medical Cannabis

**DOI:** 10.3389/fpls.2022.713481

**Published:** 2022-09-29

**Authors:** Nadav Danziger, Nirit Bernstein

**Affiliations:** Institute of Soil Water and Environmental Sciences, Volcani Center, Rishon LeZion, Israel

**Keywords:** architecture, cannabis, cannabinoids, density, stand, pruning, yield, light

## Abstract

A major challenge for utilizing cannabis for modern medicine is the spatial variability of cannabinoids in the plant, which entail differences in medical potency. Since secondary metabolism is affected by environmental conditions, a key trigger for the variability in secondary metabolites throughout the plant is variation in local micro-climates. We have, therefore, hypothesized that plant density, which is well-known to alter micro-climate in the canopy, affects spatial standardization, and concentrations of cannabinoids in cannabis plants. Canopy density is affected by shoot architecture and by plant spacing, and we have therefore evaluated the interplay between plant architecture and plant density on the standardization of the cannabinoid profile in the plant. Four plant architecture modulation treatments were employed on a drug-type medicinal cannabis cultivar, under a density of 1 or 2 plants/m^2^. The plants were cultivated in a naturally lit greenhouse with photoperiodic light supplementation. Analysis of cannabinoid concentrations at five locations throughout the plant was used to evaluate treatment effects on chemical uniformity. The results revealed an effect of plant density on cannabinoid standardization, as well as an interaction between plant density and plant architecture on the standardization of cannabinoids, thus supporting the hypothesis. Increasing planting density from 1 to 2 plants/m^2^ reduced inflorescence yield/plant, but increased yield quantity per area by 28–44% in most plant architecture treatments. The chemical response to plant density and architecture modulation was cannabinoid-specific. Concentrations of cannabinoids in axillary inflorescences from the bottom of the plants were up to 90% lower than in the apical inflorescence at the top of the plant, considerably reducing plant uniformity. Concentrations of all detected cannabinoids in these inflorescences were lower at the higher density plants; however, cannabinoid yield per cultivation area was not affected by neither architecture nor density treatments. Cannabigerolic acid (CBGA) was the cannabinoid least affected by spatial location in the plant. The morpho-physiological response of the plants to high density involved enhanced leaf drying at the bottom of the plants, increased plant elongation, and reduced cannabinoid concentrations, suggesting an involvement of chronic light deprivation at the bottom of the plants. Therefore, most importantly, under high density growth, architectural modulating treatments that facilitate increased light penetration to the bottom of the plant such as “Defoliation”, or that eliminated inflorescences development at the bottom of the plant such as removal of branches from the lower parts of the plant, increased chemical standardization. This study revealed the importance of plant density and architecture for chemical quality and standardization in drug-type medical cannabis.

## Introduction

Drug-type cannabis (*Cannabis sativa L*.) is utilized by mankind for thousands of years for religious rituals and for its' medicinal and inebriant properties (Andre et al., 2016). At the last decade, the use of cannabis sharply increased due to awareness of the plants medicinal potential and benefits for life quality, facilitated by changes in its legal status. The emerging global-markets stimulate large-scale production of cannabis, which created a need for modern agri-practices. A major challenge for quality and safe production for the pharmaceutical and recreational markets is the lack of science-based knowledge on cannabis plant biology and agronomy (Bernstein et al., 2019a).

The medical effects of cannabis are based on biologically active secondary metabolites, including terpenes, flavonoids, and cannabinoids. More than 100 cannabinoids have been identified in cannabis (Berman et al., 2018); the most abundant are the pentyl type Δ^9^-tetrahydrocannabinolic acid (THCA), cannabidiolic acid (CBDA), cannabichromenic acid (CBCA), and cannabigerolic acid (CBGA). The precursors for the cannabinoid biosynthesis are derived from the deoxyxylulose phosphate/methyl-erythritol phosphate (DOXP/MEP) pathway and the polyketide pathway (Flores-Sanches and Verpoorte, 2008). CBGA is the direct precursor for THCA, CBDA, and CBCA, and it originates from prenylation of geranyl diphosphate to olivetolic acid (Gülck and Møller, 2020). Δ^9^-tetrahydrocannabivarinic acid (THCVA) and cannabidivarinic acid (CBDVA), propyl analogs of THCA and CBDA, are minor cannabinoids originating from geranyl diphosphate and divarinic acid (Sarma et al., 2020). The biological activity is attributed to the decarboxylated forms of the cannabinoids and is affected by concentrations and interactions between cannabinoids as well as with other secondary metabolites in the plant.

Plant development and function are considerably affected by environmental conditions. Optimization of production quantity and quality, therefore, requires understanding of plant responses to environmental factors that determine the plant's phenotypic expression. Drug-type cannabis is often cultivated in greenhouses or growing rooms under environment-controlled conditions, which are needed to satisfy quality demands for the new standards defined for the highly regulated medical market (Potter, 2014). To improve growers' success and patient welfare, growing protocols that enhance yield quantity, chemical quality, and reproducibility are being developed (Bernstein et al., 2019b; Saloner et al., 2019; Eaves et al., 2020; Saloner and Bernstein, 2020, 2021, 2022a,b; Shiponi and Bernstein, 2021a,b) based on recently accumulated information on the plant responses. Recent findings demonstrate that numerous factors, including light intensity (Eaves et al., 2020; Rodriguez-Morrison et al., 2021), light quality (Magagnini et al., 2018; Danziger and Bernstein, 2021a), salt concentration (Yep et al., 2020), mineral nutrition (Saloner and Bernstein, 2021, 2022a,b; Shiponi and Bernstein, 2021b), pests and pathogens (Punja et al., 2019), affect phenotypic expression of cannabinoids in cannabis.

Plant density, or stand (Semira and Bikila, 2018), is among the main factors affecting plant development and function. It is defined as the number of plants cultivated per unit area, but could also be described by the distance, i.e., spacing between plants. Planting density affects micro-climate aspects in the plant shoot, including light availability/shading, humidity, and temperature (Yang et al., 2014). Higher plant density is therefore used to increase crop yield by increasing leaf coverage and as a result light interception (Chapepa et al., 2020). An ideal density maximizes light interception by the foliage, optimizing resource usage and growth, and too dense planting results in resource competition for light (Singh et al., 1992; Jarecki and Bobrecka-Jamro, 2011) that can compromise plant function and production.

Increased plant density was indeed documented to increase yield in a range of crops, including cotton (*Gossipium hirsutum*) (Mao et al., 2014), vine-spinach (*Basella Alba* L) (Masombo et al., 2018), and watermelon (*Citrullus lanatus*) (Akintoye et al., 2009), and the response can be cultivar-dependent (Akintoye et al., 2009). Above optimal density was reported to reduce production of individual plants as reviewed by Postma et al. (2020). One study (Campiglia et al., 2017) evaluated effects of plant density on *cannabis sativa*. It targeted industrial hemp cultivars grown for seed and stem fiber production, and reported reduced stem biomass and increased seed yield per area under increased plant density. Since industrial hemp is cultivated under different agro-techniques and density than drug-type cannabis and it targets different plant organs as yield, this information cannot directly contribute to the understanding of the drug-type crop response. Understanding responses of drug-type cannabis to plant density are needed to direct optimization of the crop morpho-development.

Plant density is known to affect the physiological and molecular state of plant tissues and therefore also primary and secondary metabolisms, and the nutritional value of crops. Various trends were noted for effects of plant density on metabolism, and increased density was reported to increase, decrease, or to have no effect on production of various metabolites. For example, carotenoid concentration of paprika (*Capsicum annuum*) (Cavero et al., 2001) and tarragon (*Artemisia dracunculus*) (Nurzyńska-Wierdak and Zawiślak, 2014) decreased with the increase in plant density; essential oil production in tarragon increased with the increase in plant density (Nurzyńska-Wierdak and Zawiślak, 2014); and in hydroponic-cultivated tomatoes (*Solanum lycopersicum*) plant spacing did not affect carotenoids, lycopene, and citric acid production (Cardoso et al., 2018). We know of no other study that evaluated effects of plant density on secondary metabolism in drug-type - medical cannabis.

Tight interrelations exist between plant spacing and shoot architecture. Architecture development of plants can be considerably affected by exogenous factors, especially microclimate parameters in the shoot. The reduced area available for the shoot under high plant density induces morphological adaptations such as elongation or retarded growth (Xiao et al., 2006). In agricultural practices, to optimize growth under higher densities, plant architecture is often altered, aiming at achieving an optimal ratio of shoot-size/yield, and reduced shading, to facilitate sufficient light penetration to the canopy (Kool, 1997; Maboko et al., 2011; Oga and Umekwe, 2016; Cardoso et al., 2018; Ayala-tafoya and Yáñez-juárez, 2019). Ideal plant density is, therefore, closely related to shoot architecture.

In the present study, we therefore focused on the interplay between plant density, plant architecture and yield quantity, and chemical standardization in medical (drug-type) cannabis. The hypotheses leading the workplan were: (i) High plant density affects chemical quality and compromises chemical uniformity in the plant, but increases inflorescence biomass per m^2^. (ii) Manipulation of the plant canopy architecture (by removal of leaves or branches, thus decreasing canopy density; or by pruning for removal of apical dominance, thus increasing branching and canopy density) affects plant responses to plant density. To test these hypotheses, we analyzed morphological, physiological, and chemical profiling of medical (drug-type) cannabis plants under two plant densities of 1 or 2 plants/m^2^ and four plant architecture manipulation treatments. The architectural treatments included defoliation, pruning and the removal of the bottom leaves, branchlets, and inflorescences, compared to a non-treated control. The study was aimed at achieving understanding required for directing horticultural practices to increase chemical quality and standardization.

## Materials and Methods

### Plant Material and Growing Conditions

A type III cultivar, i.e., containing high CDB (8–16%) and low THC (<1%) levels medical (drug-type) cannabis (*Cannabis sativa* L.) cultivar (“Topaz”, BOL Pharma, Israel), was used as a model plant in this study. The plants were propagated from cuttings, in coconut fiber plugs (Jiffy international AS, Kristiansand, Norway). The rooted cuttings were planted in 13 L pots in a peat-moss potting mixture (Kekkila-BVB, the Netherlands) and cultivated under 2 plants/m^2^. Uniform plants were randomly divided into groups of 12 plants, and each group was randomly assigned a treatment (detailed in section experimental treatments). The experiment consisted of six replicated groups per treatment. Plants of each replicated group were grown together as a single plot. The replicated plots were randomly arranged in a commercial cannabis cultivation farm in Israel (BOL Pharma, Israel), in a naturally lit greenhouse with photoperiodic light supplementation. During the vegetative stage under long photoperiod cultivation, illumination was supplied by fluorescent lamps 24 h a day. The density treatments were initiated 27 days after the rooted cuttings were transplanted to the experimental plants (during the vegetative stage). At that time, the plants were 90–100 cm in height, except the plants from the pruning treatment that due to the nature of the treatment, and were 55–65 cm shorter in height. After a total of 62 days of vegetative growth (i.e., 62 days post-transplanting), the plants were transferred to a flowering-induced short-day photoperiod of 12:12 h of light: darkness. Fertilizers were supplied by fertigation at each irrigation event, i.e., dissolved in the irrigation solution. The fertigation solution contained in ppm: N (200), P (25), K (180), Ca (30), Mg (30), S (25), Fe (0.842), Mn (0.421), Zn (0.211), Cu (0.031), Mo (0.225), and B (0.202). pH was adjusted to 6.0 with H_2_SO_4_ and the amount of S added is included in the reported concentration of S in the fertigation solution. Irrigation was supplied with 1.2 L h^−1^ discharge-regulated drippers (Plastro Gvat, Israel), four drippers per pot. The volume of irrigation water in each irrigation event was 500–800 mL pot^−1^ day^−1^, adjusted to generate ~30% of drainage. The experiment was terminated at chemical maturity of the plants, 69 days after the transfer to the short photoperiod (131 days after the rooted cuttings were transplanted to the experimental pots), following the agronomic practice for this cultivar.

### Experimental Treatments

The plants were exposed to two plant densities, and four plant architecture modulation treatments, for a total of eight treatments, in a completely randomized experimental design. The four architectural treatments studied included (i) A non-treated control [Control]; (ii) Removal of all fan leaves on the plants except very small leaves at the top of branches 3 weeks prior to harvest (69 days after the transfer to the short photoperiod), [Defoliation]; (iii) At the beginning of the vegetative growth phase (at the time of transplanting), the top of the rooted cuttings was pruned, leaving the six bottom branches [Pruning]; (iv) Removal of the branches and leaves from the lower one-third part of the plants at the transition to the short photoperiod, 62 days post-transplanting (we named this treatment “Bottom branches and leaves removal”) [BBLR]; this treatment is also known as “Lollipoping” in the cannabis industry jargon. Plants of each architecture treatment were evaluated under two plant densities of either 1 or 2 plants/m^2^. The plants in each replicated plot were arranged in four rows with three plants per row, and a central plant from a central rows was used for the measurements. The remaining plants in the plot received the same treatment and served as margins.

### Plant Growth and Development

Plant height was measured non-destructively biweekly as the difference from the plant base to the top of the apical meristem on the main stem, or on the tallest branch in the pruning treatments. Stem diameter was measured with a digital caliper (Signet tools international co., LTD., Shengang District, Taiwan), at the middle of the first internode from the plant base. Fresh biomass of inflorescences, stems, and fan leaves was measured for each plant by destructive sampling at the termination of the experiment. Inflorescences were trimmed by an industrial trimmer T2 twister (Keirton inc. Ferndale, WA, USA) and the trimmed inflorescences were weighted again for the calculation of the trimmed inflorescence leaves biomass. Dry inflorescences yield was determined following drying in the dark for 20 days at 45% air humidity and 19°C, to ~10% humidity.

### Physiological Responses

The measurements were conducted 1 week after the initiation of the leaf removal treatment (2 weeks prior to harvest), i.e., 69 days after the transfer to the short photoperiod. Following the experimental design, all measurements were conducted with six biological repeats (i.e., for six plants).

#### Pigment Concentrations, Gas Exchange Parameters, Water Use Efficiency, and PAR

Concentrations of the photosynthetic pigments chlorophyll *a*, chlorophyll *b*, and carotenoids were measured as previously described (Ignat et al., 2013; Saloner et al., 2019). In short, five discs with diameter of 0.6 cm were severed from the youngest mature leaf on the main stem (or alternatively from the highest primary branch in the pruning treatments) after it was washed twice in distilled water and blotted dry. Pigment extraction was conducted as described by Gorelick et al. (2015), and pigment concentrations were calculated according to Lichtenthaler and Wellburn (1983).

Stomatal conductance, photosynthesis and transpiration rates, and intercellular CO_2_ concentration were measured with LI-COR 6400XT (LI-COR, Lincoln, NE, USA). The measurements were performed on the youngest mature leaf on the main stem (or alternatively on the highest primary branch in the pruning treatments), at 8–10 am [CO_2_ concentration: 400 mgL^−1^ and PPFD: 500 μmol (m^2^s)^−1^]. Leaves temperature was kept at 25°C and relative humidity at 60%. Water use efficiency (WUE) was calculated from Equation 1.


(1) $$\begin{array}{r} Water\;use\;efficieancy\;\left(\%\right)=\frac{Photosynthesys\;rate}{Transpiration\;rate}*100 \end{array}$$

Photosynthetic active radiation (PAR) was measured at four heights along the plant (0, 0.5, 1.2, and 2 m from the plant base) using an Apogee quantum sensor MQ-500 (Apogee Instruments, Logan, UT, USA).

#### Membrane Leakage and Osmotic Potential

The youngest mature leaf on the main stem (or on the tallest branch in the pruning treatments) was washed twice in distilled water and blotted dry. For membrane leakage analysis, the middle leaflet was then separated and submerged in 30 mL of double distilled water. After 24 h of shaking in a horizontal shaker, electric conductivity (EC) of the sample was measured using an EC-meter (Cyberscan CON 1500, Eutech Instruments Europe B.V., Nijkerk, The Netherlands). Following autoclaving (30 min at 121°C) (Shoresh et al., 2011) and 30 min of cooling at room temperature, EC was measured again. Membrane leakage was calculated as the percentage of the first EC measurement value from the value of the second measurement (Kravchik and Bernstein, 2013).

For osmotic potential measurements, ~150 mg of leaf tissue was inserted into a 1.7-mL Eppendorf tube and immediately frozen in liquid N and kept in −20°C until further analysis. For expression of the cell-sap from the tissue, the sample was partially thawed and macerated inside the tube with a pestle and centrifuged (Sigma Laboratory Centrifuges, Germany) at 4°C and 6,000 rpm for 5 min. A 50 μL aliquot of the supernatant was measured in a cryo-osmometer (Gonotec, Berlin, Germany) to determine the osmotic potential of the leaf tissue sap.

### Cannabinoid Analyses

For evaluation of the effect of the treatments on the cannabinoid profile and its' standardization in the plant, inflorescences were sampled for cannabinoid analyses from five locals along the plants, illustrated in Supplementary Figure 1: (1) The top most inflorescence; (2) The apical inflorescence of a high branch (the 4th highest branch); (3) The apical inflorescence of a low branch (4th from plant base); (4) An inflorescence located close to the stem (an axillary inflorescence) at the top area of the plant (2nd branch from the top); (5) The bottom most inflorescence located closest to the stem (an axillary inflorescent, from the 1st branch from the plant base). Trimmed inflorescences were dried in the dark for 20 days at 45% air humidity and 19°C to 10% humidity in an environment-controlled chamber. Cannabinoid analysis was conducted for six replicated plants per treatment.

The dried inflorescences were ground using a manual herb grinder. Fifty mg of the ground tissue was placed with 10 mL of ethanol in a 20-mL glass vial and was shaken in a reciprocal shaker for 1 h at room temperature. The extract was filtered through PVDF (a polyvinylidene difluoride membrane filter) of 0.22-μm pore size (Bar-Naor ltd, Ramat Gan, Israel). Concentrations of cannabinoids in the filtered extracts were analyzed with a Jasco 2000 Plus series HPLC system that consist of an autosampler, column compartment, quaternary pump, and a PDA detector (Jasco, Tokyo, Japan). Chromatographic separation was performed with a Luna Omega 3 μm Polar C18 column (Phenomenex, Torrance, CA USA) with acetonitrile: water 75:25 (v/v) with 0.1% (v/v) formic acid, at the isocratic mode. The flow rate was 1.0 mL min^−1^. Calculation of cannabinoid concentrations was based on pure analytical standards that were purchased from Sigma-Aldrich (Germany): cannabichromene (CBC), cannabichromenic acid (CBCA), cannabichromevarin (CBCV), cannabigerol (CBG), cannabigerolic acid (CBGA), cannabinol (CBN), cannabinolic acid (CBNA), cannabidiol (CBD), cannabidiolic acid (CBDA), cannabicyclol (CBL), cannabidivarin (CBDV), cannabidivarinic acid (CBDVA), Δ^9^-tetrahydrocannabivarinic acid (THCVA); from Cayman chemical company (Pennsylvania, USA) cannabicitran (CBT); and from Restek (Pennsylvania, USA) Δ^9^-tetrahydrocannabinolic acid (THCA), Δ^9^-tetrahydrocannabinol (THC), Δ^8^-tetrahydrocannabinol (Δ^8^-THC), Δ^9^-tetrahydrocannabivarin (THCV). *R*^2^ values for linear regressions of the calibrations curves of all cannabinoid standards were >0.994 (Saloner and Bernstein, 2021); Concentrations of CBD were small (<0.21%) and are, therefore, presented together with the CBDA concentrations. Concentrations of CBC, CBG, CBN, CBNA, CBL, CBDV, CBT, THC, Δ^8^-THC, and THCV were lower than the detection limits. Cannabinoid yield per cultivation area (mg/m^2^) was calculated from the plant average concentration of the cannabinoids.

### Evaluation of Spatial Uniformity of the Cannabinoid Profile in the Plant

Two scores were developed to evaluate the uniformity of cannabinoid concentrations within a plant: “Cannabinoid Variation Score” (CVS) evaluates the variability of an individual cannabinoid in the plant, and “Plant Variation Score” (PVS) evaluates an integration of variability of all identified cannabinoids in the plant. These scores were developed from two indexes (“Cannabis uniformity” and “plant uniformity score”) that were suggested and applied by Danziger and Bernstein (2021b) for the evaluation of treatments' effects on uniformity of compounds in plants. The evaluation is based on the enumeration of the percentage of inflorescences in a treatment having a concentration of a secondary metabolite that varies by more than a defined percentage from the plant average concentration. In the present study, we used variation of 15% for the calculations of CVS. For the calculations, first, the average concentration of each identified cannabinoid (CAC) is calculated as of Equation 2 (30 samples were used for the calculations). Second, the concentration of the cannabinoid in each sample was compared to the generated average, and the number of samples that varied by more than 15% from the average were counted (denotes by the numerator in Equation 3). This number was divided by the number of samples which contained the specific cannabinoid (as not all samples had detectable concentrations of all cannabinoids) and multiplied by 100 to receive the CVS value (Equation 3). The CSV value, therefore, has units of %; it is the percentage of samples with a concentration of a specific cannabinoid varying by up to 15% from the average. It, therefore, represents variability for a specific cannabinoid (or any other evaluated plant compound). In order to receive an integrated value for uniformity of all the identified cannabinoids, the calculated CVS values for all individual cannabinoids were averaged to receive the PVS (Equation 4). The higher the PVS score, the more variable is the treatment. These variation scores can be applied for the evaluation of uniformity in the plant concentrations of secondary metabolites, as well as other chemical compounds.


(2) $$Cannabinoid\;average\;concentration\;(CAC)=\frac{\sum^{\text{​}}cannabinoid\;concentration\;in\;the\;individual\;samples\;}{No.\;of\;samples\;that\;contained\;the\;cannabinoid}*100$$

(3) $$Cannabinoid\;Variation\;Score(CVS)\;[\%]=\frac{\left|(No.\;of\;samples\;with\;conc.<CAC*0.85)\;{\cup^{\text{​}}}^{\text{​}}\;(No.\;of\;samples\;with\;conc.>CAC*1.15)\right|}{No.\;of\;samples\;that\;contained\;the\;identified\;cannabinoid}*100$$

(4) $$Plant\;Variation\;Score\;(PVS)[\%]=\frac{\sum^{\text{​}}CVS\;for\;each\;of\;the\;identified\;cannabinoids}{The\;number\;of\;identified\;cannabinoids}$$

### Statistical Analysis

The data were subjected to a one-way and two-way analysis of variance (ANOVA) (α < 0.05) followed by Tukey's HSD *post-hoc* test. The data met the assumption of homogeneity of variances. Comparison of relevant means was performed using Fisher's LSD test at 5% level of significance. The analysis was performed with the Jump software (version 9, SAS 2015, Cary, NC, USA).

## Results

### Plant Development

**Figure 1:**
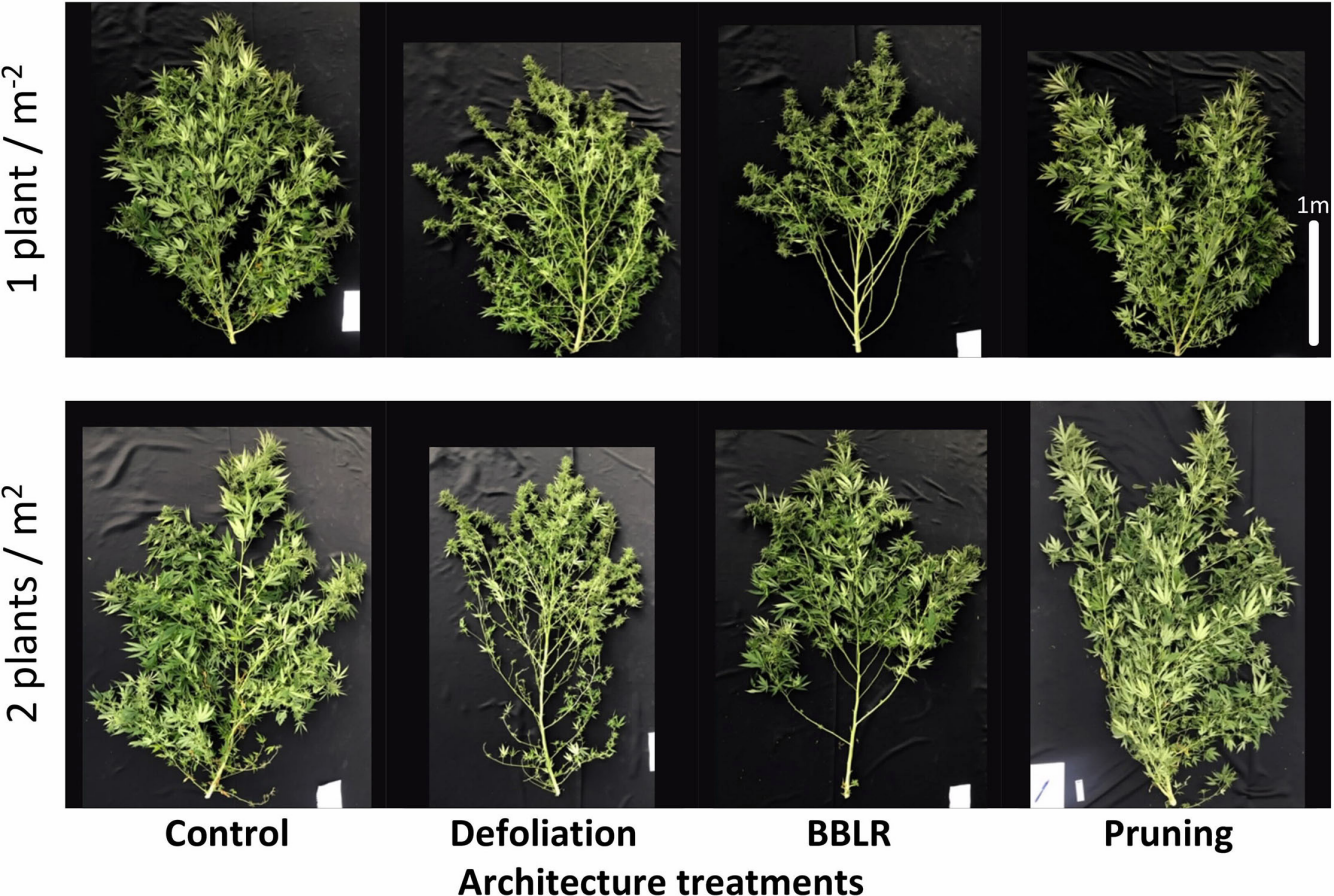
Effect of planting density (1 or 2 plants m^−2^) on visual appearance of medical cannabis plants, subjected to four plant architecture modulation treatments. Control, Defoliation, removal of leaves and branches from the bottom part of the plant [BBLR] at the transition to the flowering stage, and pruning at the beginning of the vegetative growth stage. The images were taken 69 days following the transition to the short photoperiod, at the time of harvest.

**Figure 2:**
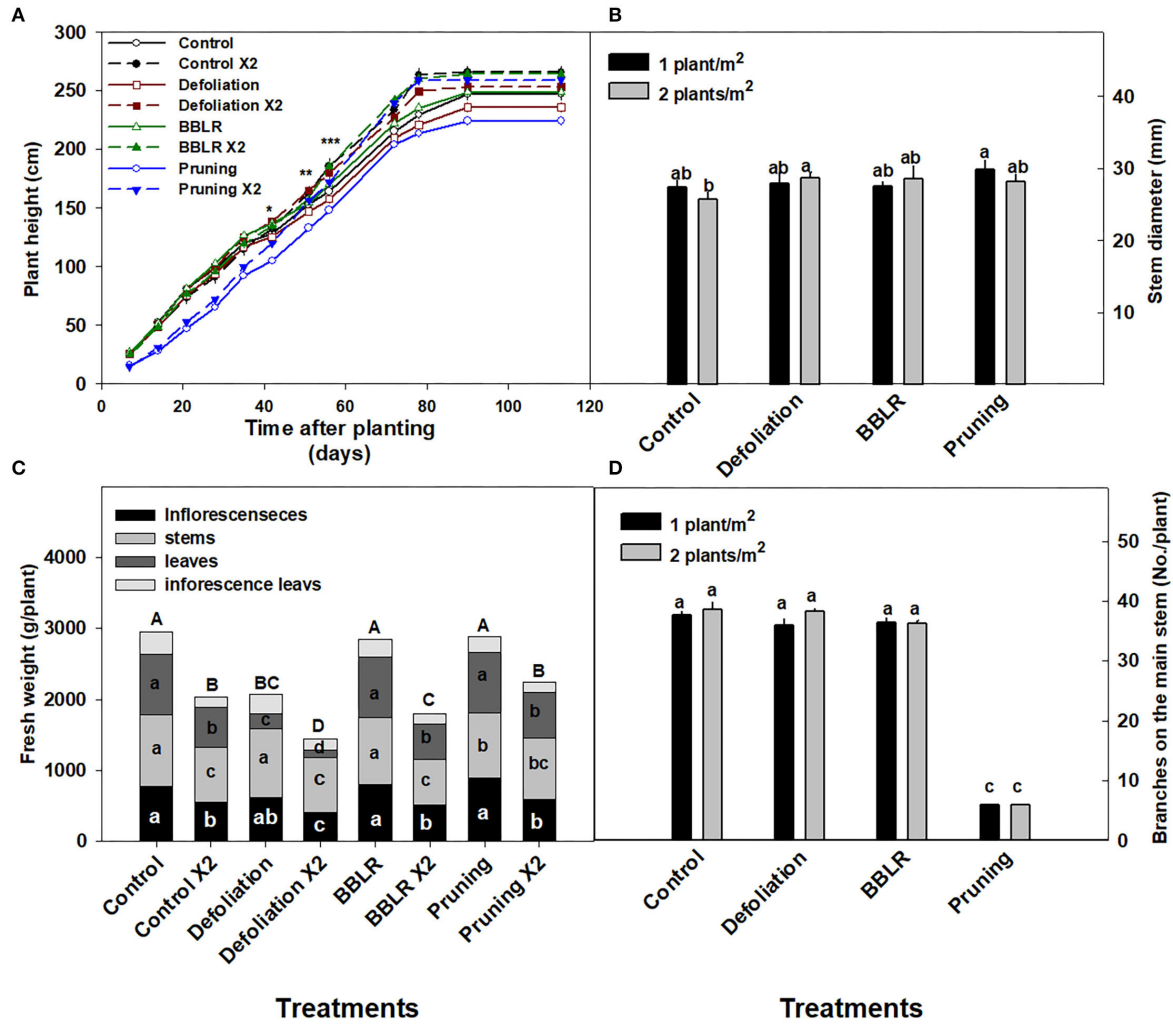
Effects of plant density and architecture modulation treatments on plant morphology. Plant height **(A)**, biomass of plant organs and of the whole plant (bars) **(B)**, Stem diameter **(C)**, and number of branches **(D)** at harvest. The results are mean (*n* = 6) and SE (in **C,D**). Different letters above the bars represent significant differences between treatments by Tukey HSD test at α = 0.05. In **(A)**, asterisks represent the day significant differences were first identified between density treatments within an architecture treatment (*-“Pruning”, **-“Control,” and “Defoliation,” ***-“BBLR”). In **(B)**, different letters within the bars across a plant organ, represent significant differences between treatments. x 2 represents the density of 2 plants/m^2^.

Plant density as well as the architectural manipulation treatments affected plant development and the visual appearance of the plants (Figure 1). Plants from the higher density treatments were taller and slightly narrower in appearance compared to the plants from the lower density treatments. The “BBLR” plants had no leaves or inflorescences at the bottom part of the plant since these were removed as part of the treatment, and the “Pruning” treatment caused the plant to develop two main stalks rather than the natural one main stem form. Additionally, in the “Control” and the “Defoliation” treatments, lower leaves and branches in the higher density treatment (2 plants/m^2^) were senescing. The taller stature of the higher density plants is also seen in Figure 2A that shows the plants of all high-density treatments were significantly higher compared with their low-density counterparts.

Fresh weight of the plant was significantly reduced by the increase in plant density across all the architecture treatments, with a 22–37 and 28–36% decrease in total plant fresh weight, and inflorescence yield, respectively (Figure 2B). The least-affected organ was the stem, with a 5–32% less fresh weight compared with the lower density treatments, whereas both fan leaves and inflorescence leaves were highly susceptible to planting density, demonstrating 48–74% fresh weight compared with the low-density treatments. In the “Defoliation” treatments as well, that involve an inherent reduction of leaf tissue biomass, a significant reduction in leaf tissue biomass was induced by the increase in cultivation density.

The diameter of the stem (Figure 2C) was not affected significantly by plant density; and neither did the number of branches that were developed on the plants (Figure 2D). The number of branches on the main stem was significantly lower for the “pruning” treatments compared with all other treatments (Figure 2D), representing the six bottom branches that were kept on the plants during decapitation. Following the decapitation, the plant body developed mainly from two main stalks (i.e., secondary branches) (Figure 1).

Inflorescence yield production per cultivation area (gDW/m^2^) was higher (by 28–78%) in the higher density treatment than in the lower density treatments in the control, “BBLR” and “defoliation” treatments, but was not significantly affected by plant density in the “Pruning” treatment (Table 1).

**Table 1:** Effect of plant density on inflorescence yield per cultivation area (g DW/m^2^)^B^.

**Treatment**	**1 plant/m^**2**^**	**2 plants/m^**2**^**
Control	222 ± 14.9^b^	321 ± 19.3^a^
Defoliation	180 ± 17.3^b^	320 ± 29.3^a^
BBLR^A^	229 ± 12.5^b^	295 ± 27.3^a^
Pruning	255 ± 10.6^a^	232 ± 12.9^a^

A *Removal of branches and leaves from the bottom of the plant*.

B *Different lowercase letters by the averages within a row signifies significant differences between the two density treatments according to Tukey HSD test, at α = 0.05 (n = 6)*.

### Chemical Profile

Cannabinoid concentrations were determined in five defined locals in the plants, including (1) The top most inflorescence; (2) The apical inflorescence of a high branch (the 4th highest branch); (3) Apical inflorescence of a low branch (4th from plant base); (4) An axillary inflorescence located close to the stem at the top area of the plant (2nd branch from the top); (5) The bottom most inflorescence located closest to the stem (an axillary inflorescence from the lowest branch closest to the stem (Supplementary Figure 1).

**Figure 3:**
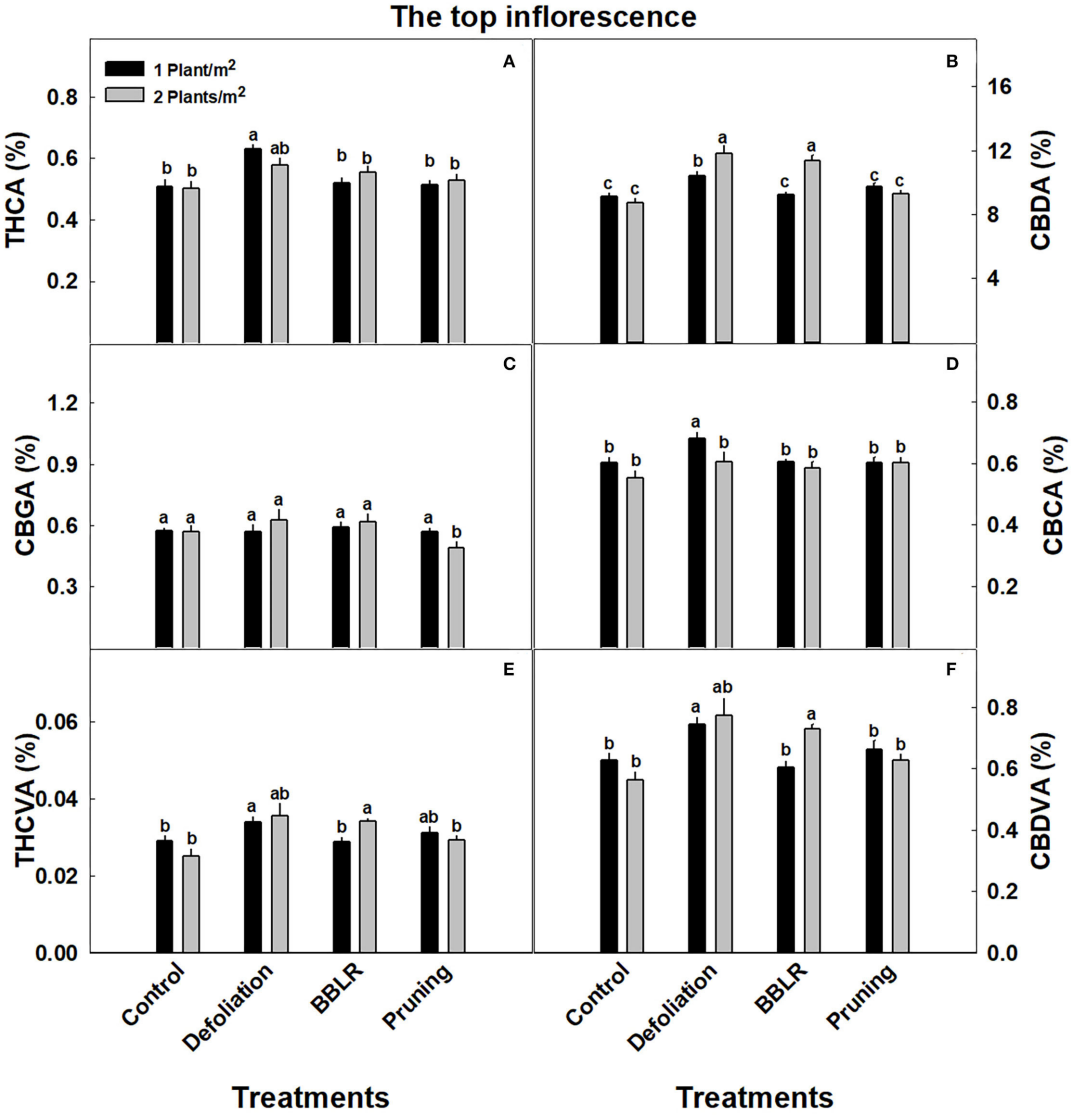
Cannabinoid concentrations at the top inflorescence of cannabis plants grown under two densities and four architectural modulation treatments. The results are means and SE (*n* = 6). THCA **(A)**, CBDA **(B)**, CBGA **(C)**, CBCA **(D)**, THCVA **(E)**, and CBDVA **(F)**. Different letters above the bars represent significant differences between treatments by Tukey HSD test at α = 0.05. “BBLB” removal of leaves and branches from the bottom part of the plant.

**Figure 4:**
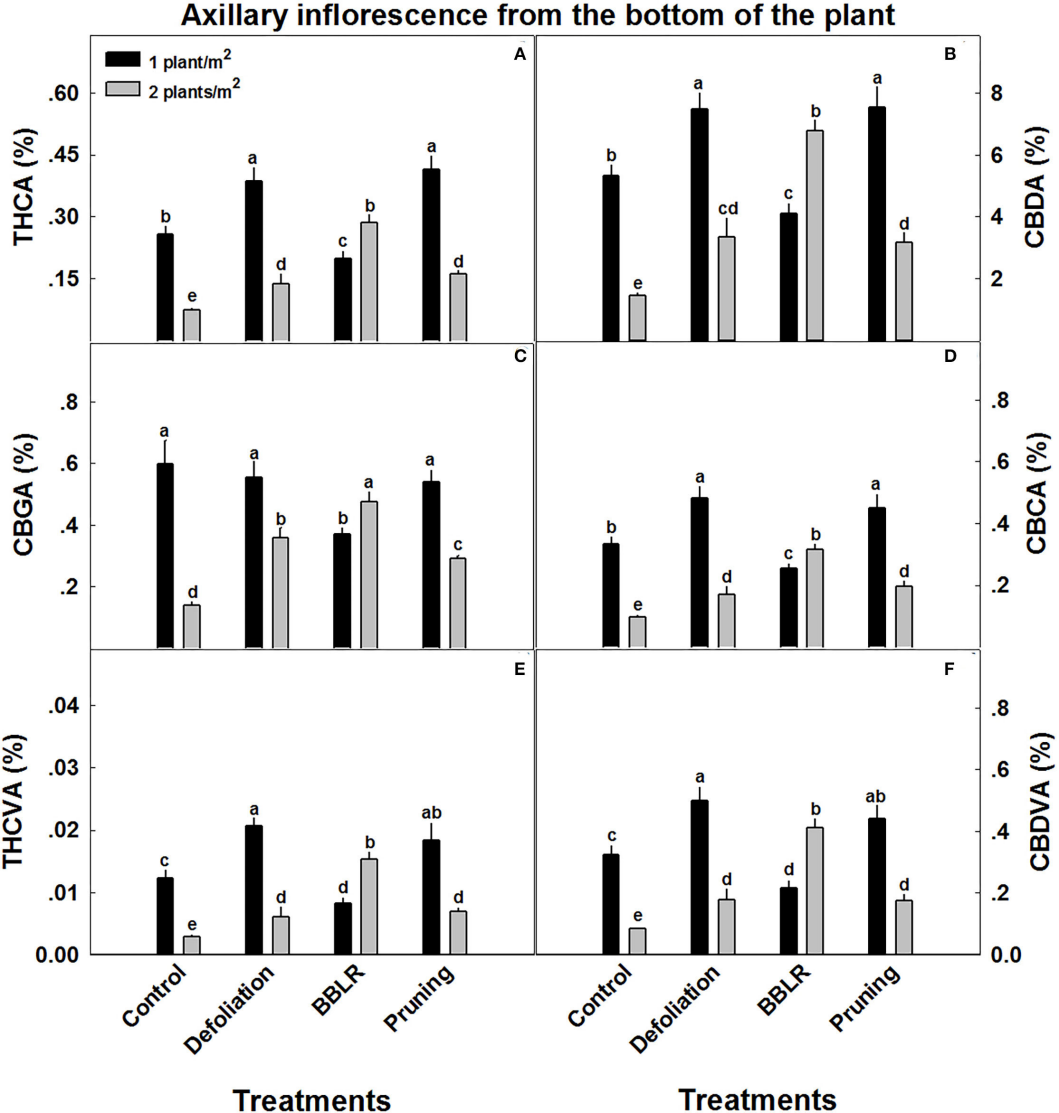
Cannabinoid concentrations in axillary inflorescences from the lowest branch on the plant, close to the point of emergence from the main stem. The cannabis plants were grown under two densities (1 or 2 plants m^−2^) and four architectural modulation treatments. Cannabinoids detected include THCA **(A)**, CBDA **(B)**, CBGA **(C)**, CBCA **(D)**, THCVA **(E)**, and CBDVA **(F)**. The results are mean and SE (*n* = 6). Different letters above the bars represent significant differences between treatments by Tukey HSD test at α = 0.05. “BBLB” removal of leaves and branches from the bottom part of the plant.

Cannabinoid concentrations in the highest inflorescence on the plant (location 1), which is the representative inflorescence commonly sampled for cannabinoid analysis, are presented in Figure 3. The concentrations in this location were overall not affected by the treatments, with only small changes (*p* <0.05) induced by some treatments. Specifically, CBDA (Figure 3B) levels were higher in the closely spaced plants of the “BBLR” and “Defoliation” treatments compared with the less dense treatments; and under the high density, CBDA concentrations in these treatments were also higher than in the “Control” and “Pruning” treatments. At the lower density, THCA (Figure 3A) and CBCA (Figure 3D) levels were higher in the “Defoliation” treatment compared with all other treatments, and CBCA of the defoliation low-density treatment was higher than all other architecture treatments also under the higher density. The concentration of CBGA (Figure 3C), the precursor of all the above-mentioned cannabinoids, was similar across all treatments except for the “Pruning” higher density treatment that had a lower concentration. For both THCVA and CBDVA, no significant changes between the density treatments were seen except for the “BBLR” treatment that had higher levels in the high-density plants (Figures 4E,F).

Unlike the inflorescences from the top of the plant from location 1, cannabinoid concentrations in axillary inflorescences from low branches of location 5 (Figure 4) were considerably affected by plant density. Several trends were observed: (i) Most important is the overall decrease in cannabinoid concentrations, up to 90% reduction compared with the top inflorescence. (ii) In all treatments, except “BBLR,” concentrations of all detected cannabinoids were considerably lower in the higher density treatment. The treatment that was affected the most by plant density is the “Control,” with a decline of 71–76% in the concentrations of all six detected cannabinoids with the increase in plant density. (iii) In the “BBLR” treatment, cannabinoid concentrations were 25–90% higher in the high-density plants. (iv) In this location (location 5), concentrations of all identified cannabinoids, except for CBGA, were lower in the “Control” treatment than in both the “Pruning” and “Defoliation” treatments when comparing similar densities. Cannabinoid yield per cultivation area (mg/m^2^) were not affected by neither architecture nor density treatments (Table 2).

**Table 2:** Cannabinoid yield per cultivation area (mg/m^2^) as affected by plant density and architecture modulation treatments^B^.

	**CBDVA (mg/m^**2**^)**	**CBGA (mg/m^**2**^)**	**THCVA (mg/m^**2**^)**	**THCA (mg/m^**2**^)**	**CBCA (mg/m^**2**^)**	**CBDA (mg/m^**2**^)**
**Treatment**	**1 plant /m** ^**2**^					
Control	0.94 ± 0.06^a^	0.93 ± 0.06^a^	0.043 ± 0.003^a^	0.80 ± 0.04^a^	0.95 ± 0.05^a^	14.27 ± 0.79^a^
Defoliation	0.90 ± 0.10^a^	0.79 ± 0.10^a^	0.041 ± 0.004^a^	0.76 ± 0.08^a^	0.87 ± 0.09^a^	13.31 ± 1.40^a^
BBLR^A^	0.91 ± 0.08^a^	0.92 ± 0.08^a^	0.042 ± 0.004^a^	0.79 ± 0.07^a^	0.93 ± 0.07^a^	13.99 ± 1.08^a^
Pruning	1.08 ± 0.05^a^	0.94 ± 0.03^a^	0.050 ± 0.002^a^	0.87 ± 0.04^a^	1.01 ± 0.05^a^	16.51 ± 0.78^a^
**Treatment**	**2 plant /m** ^**2**^					
Control	1.02 ± 0.19^a^	0.94 ± 0.17^a^	0.048 ± 0.009^a^	0.85 ± 0.15^a^	0.98 ± 0.17^a^	15.08 ± 2.64^a^
Defoliation	1.03 ± 0.21^a^	0.94 ± 0.17^a^	0.047 ± 0.009^a^	0.73 ± 0.12^a^	0.80 ± 0.13^a^	15.72 ± 2.55^a^
BBLR^A^	1.24 ± 0.16^a^	1.07 ± 0.14^a^	0.055 ± 0.007^a^	0.87 ± 0.12^a^	0.95 ± 0.13^a^	19.04 ± 2.62^a^
Pruning	1.00 ± 0.19^a^	0.89 ± 0.17^a^	0.046 ± 0.009^a^	0.84 ± 0.16^a^	0.98 ± 0.19^a^	15.31 ± 3.00^a^

A *Removal of branches and leaves from the bottom of the plant*.

B *Data followed by the same small letter within a column that includes both density treatments, signifies that the cannabinoid concentration did not differ significantly between treatments according to Tukey HSD test, at α = 0.05 (n = 5)*.

**Figure 5:**
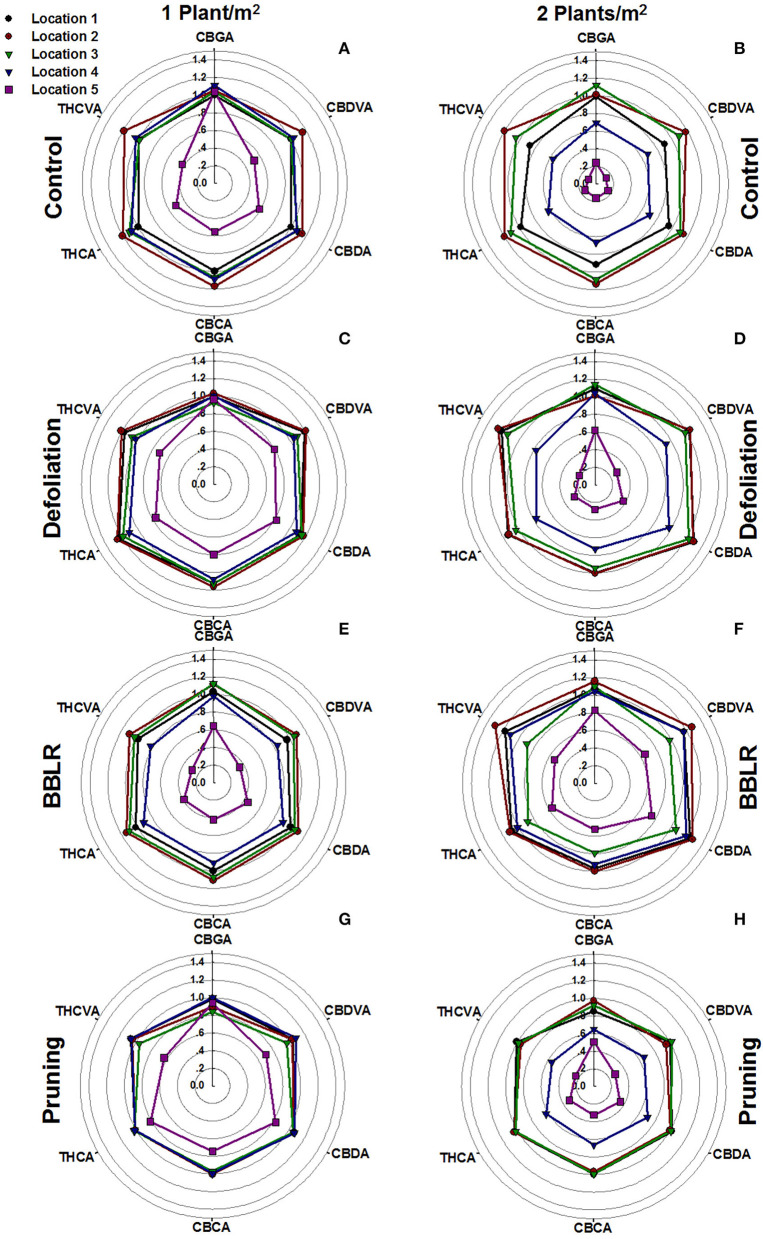
Effect of plant density and plant architecture treatments on cannabinoid concentrations in inflorescences from five locations in the plant. The data presented are concentration of each cannabinoid, relative to its concentration in the apical inflorescence of the main stem of the “Control” at the 1 plant/m^2^ treatment (location1). 1 plant/m^2^ density **(A,C,E,G)**, 2 plants/m^2^ density **(B,D,F,H)**. Architecture manipulation treatments: “Control” **(A,B)**, “Defoliation” **(C,D)**, Removal of leaves and branches from the bottom part of the plant [“BBLR”] **(E,F)** and “Pruning” **(G,H)**. The results are mean (*n* = 6).

To visualize chemical uniformity across the plant, the concentrations of each cannabinoid at the five evaluated locations throughout the plant were divided by the concentrations in location 1 of the “Control” treatment (the highest inflorescence on the plant) under the 1 plant/m^2^ density. These ratios were plotted to a radar chart (Figure 5). This normalization facilitates comparison of trends between locations, and across treatments that are presented in the sub-charts of Figure 5. The results reveal three major trends: (i) Axillary inflorescences from the bottom of the plants (location 5) accumulated significantly lower concentrations of cannabinoids across all treatments. (ii) For three treatments “Control” (Figure 5B), “Defoliation” (Figure 5D), and “Pruning” (Figure 5H), the double density hampered cannabinoid synthesis at location 4 (an inner axillary inflorescence at the top part of the plant). (iii) Treatments effects on specific cannabinoids. The outer perimeter shape of each radar chart represents the chemical profile, and a “misshaped” hexagon, therefore, indicates a change in ratios between all cannabinoids. For example, in all treatments (Figures 5A–H), the CBGA corner of location 5 is closer to the outer perimeter than the corners of all other cannabinoids showing that CBGA is the cannabinoid least affected by the spatial location.

To further evaluate how spatial uniformity of cannabinoid concentration in the plants was affected by the treatments, an index previously developed by Danziger and Bernstein (2021b) was used to rate plant uniformity by comparing each inflorescence to the plant average, allowing various rates of deviation from it (Table 3). Both the “*Plant Variation Score*” and the “*Cannabinoid Variation Score*” were higher in the densely grown plants for all cannabinoids and under all levels of acceptance (with the exception of CBGA of BBLR), indicating that higher density impairs cannabinoid uniformity under these growing conditions. For the higher plant density, at all acceptance rates (excluding 5%), the plant variation score of “BBLR” was lowest demonstrating the best chemical uniformity, and “Control” was ranked to have the lowest chemical uniformity.

**Table 3:** Effect of plant density (1 and 2 plants/m^2^) and architectural modulation treatments on chemical uniformity of cannabinoids in *Cannabis sativa* plants^D^.

	**Cannabinoid variation score (%)^A^**						**Plant variation score (%)^B^**				
	**CBDVA**	**CBGA**	**THCVA**	**THCA**	**CBCA**	**CBDA**	**5%**	**10%**	**15%**	**25%**	**50%**
**Treatment**	**1 plant /m** ^**2**^										
Control	37^f^	40^d^	43^f^	47^e^	40^e^	33^e^	79^c^	59^e^	41^d^	22^e^	^e^
Defoliation	43^e^	27^e^	47^e^	43^e^	43^de^	33^e^	77^d^	56^f^	39^e^	18^f^	2^e^
BBLR^C^	47^d^	53^b^	57^d^	53^d^	47^d^	40^d^	79^c^	66^d^	49^d^	30^d^	8^d^
Pruning	43^e^	27^e^	47^e^	33^f^	40^e^	37^d^	77^d^	54^f^	38^e^	17^f^	4^e^
	**2 plants /m** ^**2**^										
Control	76^a^	72^c^	76^b^	90^a^	76^a^	72^a^	95^a^	87^a^	77^a^	60^a^	19^a^
Defoliation	73^a^	40^d^	77^b^	70^c^	70^b^	67^ab^	88^b^	76^b^	66^b^	49^b^	17^b^
BBLR	60^c^	47^c^	73^c^	80^b^	60^c^	43^c^	94^a^	73^c^	61^c^	38^c^	9^d^
Pruning	66^b^	72^a^	83^a^	69^c^	66^bc^	62^b^	90^b^	80^b^	70^b^	43^b^	13^c^

A *“Cannabinoid variation score” represents the percentage of inflorescences deviating by more than 15% from the average cannabinoid concentration in the plant*.

B *“Plant variation score” represent the percentage of inflorescences having concentrations similar to the treatment average across all cannabinoids. It is presented for five levels of variation acceptance: 5, 10, 15, 25, and 50% variation from the treatments average*.

C *“BBLB”-removal of leaves and branches from the bottom part of the plant*.

D *Different lowercase letters near the means within a column represent significant differences between treatments for each cannabinoid by Tukey HSD test, α = 0.05*.

### Physiological Response

**Figure 6:**
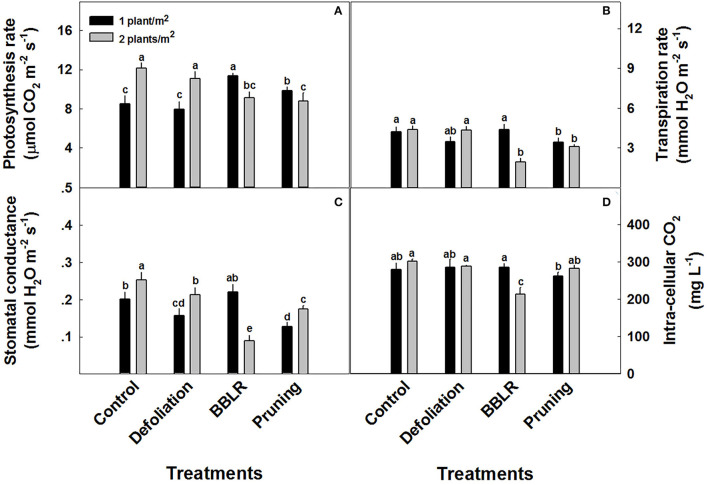
Response of gas-exchange parameters to planting density (1 and 2 plants/m^2^) and plant architecture treatments in medical cannabis plants. Photosynthesis **(A)**, Stomatal conductance **(B)**, Transpiration **(C)**, and intracellular CO_2_ **(D)**. The results are mean and SE (*n* = 6). Different letters above the bars represent significant differences between treatments by Tukey HSD test at α = 0.05.

Plant gas-exchange parameters of the youngest mature leaf on the main stem (or alternatively on the highest primary branch in the pruning treatments) were significantly affected by both the architecture modulation treatments and plant density (Figure 6). Increased density stimulated photosynthesis and stomatal conductance in both “Control” and “Defoliation” plants (Figures 6A,C), but reduced photosynthesis and stomatal conductance in “BBLR” plants and photosynthesis in pruned plants. Transpiration rate (Figure 6B) was unaffected by plant density except for an inhibition in the double density “BBLR” treatment, which showed 55% decline compared with the less dense treatment. The reduced transpiration in this treatment, which implies reduced stomatal opening, reduced also tissue aeration and concentration of CO_2_ in the leaf mesophyll (Figure 6D).

**Figure 7:**
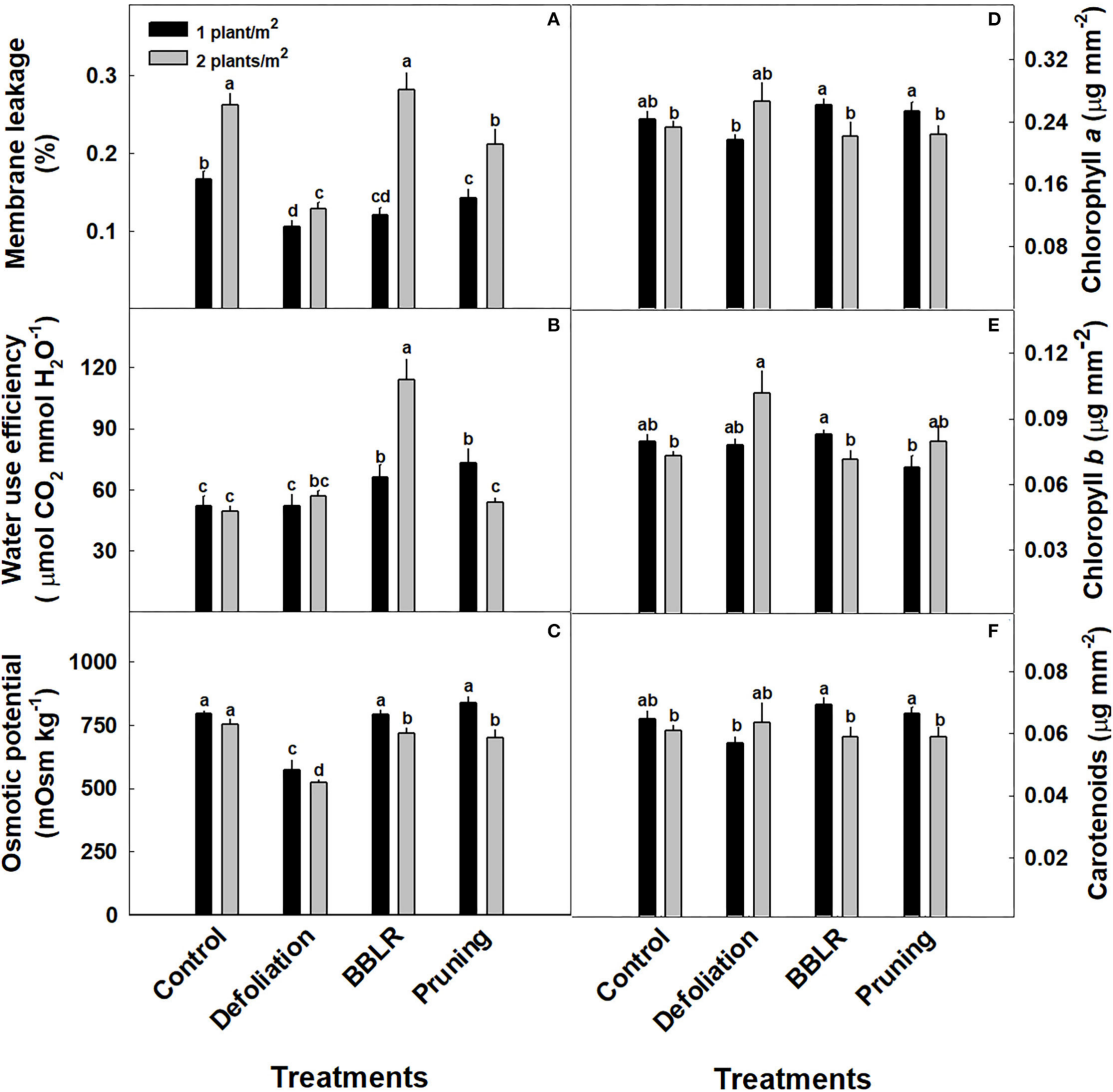
Effects of plant density (1 or 2 plants/m^2^) and plant architecture modulating treatments on medical cannabis plants. Membrane leakage **(A)**, Water use efficiency **(B)**, Osmotic potential **(C)**, Chlorophyll *a* **(D)**, Chlorophyll *b* **(E)**, carotenoids **(F)**. The results are mean and SE (*n* = 6). Different letters above the bars represent significant differences between treatments by Tukey HSD test at α = 0.05.

The plants water management strategy was measured using two indicators (Figure 7): Water Use Efficiency (WUE) and osmotic potential; and membrane leakage was measured as an indicator of plant stress representing cell membrane damage. Under all architecture altering treatments, membrane leakage was higher under higher density (Figure 7A), and under the lower density all plant architecture treatments showed a lower stress response than the “Control.” The plants WUE (Figure 7B) was calculated using the CO_2_ assimilation rate, and it presents three different responses according to the plant architecture treatments: higher density increased WUE in the “BBLR” treatments, reduced WUE in the “Pruning” treatment but had no effect in both the “Control” and “Defoliation” treatments. The osmotic potential (Figure 7C) was affected by both plant density and plant architecture modulation treatments. In the “Defoliation,” “BBLR,” and “Pruning” plants, the osmotic potential was lower under higher density, whereas no difference was seen in the “Control.” In addition, “Defoliation” reduced the osmotic potential compared to the “Control.”

Overall, the effects of the treatments on accumulation of photosynthetic pigments were small, with some statistically significant trends (Figures 7D–F). Pigment accumulation had a varied response to plant density (Figures 7D–F). For both “Control” and “Defoliation,” no difference between densities was apparent in neither chlorophyll *a*, chlorophyll *b* nor carotenoids. However, “BBLR” and “Pruning” plants usually had higher pigment concentrations in the low-density plants (Figures 7D,F). Plant architecture and planting density affected light penetration to the plant (Supplementary Figure 3), and under all plant architecture treatments, increasing density reduced light penetrance. Light intensity along the plants in both defoliation-density treatments was higher for all other treatments (Supplementary Figure 3).

## Discussion

Cultivation and environmental conditions considerably affect secondary metabolism in plants, which is of importance for the medical and recreational product of drug-type cannabis (Gorelick and Bernstein, 2017). In the fast-growing world of cannabis pharmaceuticals, agronomic knowhow for production of high-quality, safe, and chemically standardized plant material needs to rapidly develop. To keep up with demand, various agricultural practices are used by the growers, but the effects of these newly adopted cultivation practices on product quality were usually not tested. Some agronomic practices such as mineral nutrition (Bernstein et al., 2019b; Bevan et al., 2021; Saloner and Bernstein, 2021, 2022a,b; Shiponi and Bernstein, 2021b), light quality (Magagnini et al., 2018; Danziger and Bernstein, 2021a; Westmoreland et al., 2021), light intensity (Potter and Duncombe, 2012), and manipulation of the canopy architecture (Danziger and Bernstein, 2021b,c) were recently shown to change yield quantity and chemical quality in drug-type medical cannabis, and to affect the physiological state of the plant. Spatial variabilities in environmental conditions within the canopy are directly related to canopy density via effects on shading and air circulation (Morales et al., 2000; Boulard et al., 2017) and are considered to be a key to the lack of chemical standardization in cannabis cultivation. Crop plants depend on light radiation for their growth and development and hence for yield production (Yang et al., 2014). Plant density and plant architecture affect light penetration through the canopy and are, therefore, important crop growth parameters. A common method to increase yield per cultivation area is to increase plant stand, i.e., to grow under higher densities (Bekhradi et al., 2014; Nurzyńska-Wierdak and Zawiślak, 2014). An increase of plant density changes numerous micro-climatic conditions in the plant canopy, which can alter floral development and chemical profile (Khorshidi et al., 2009; El-Zaeddi et al., 2016). We have, therefore, hypothesized that the concentrations and spatial standardization of cannabinoids in drug-type cannabis plants could be affected by plant density, and that the response will be an interplay with architecture manipulations. The results identified that the cannabinoids profile is indeed highly affected by plant density and by architectural manipulations thus supporting the hypothesis; and furthermore, highlighting the importance of plant density and canopy structure for the standardization of the chemical profile. Our results thus expand the ability to regulate cannabinoid metabolism and yield in medical cannabis, and therefore direct researchers and growers to improve the chemical quality.

### Yield and Yield Components

Cannabis-based therapeutics use inflorescences or their extracts for patients' care, and the cannabis inflorescence is the marketable yield in medical cannabis. A wide range of cultivation practices is utilized in the production industry, and cultivation is based on growth of plants that vary dramatically in size, architecture, and plant density. For economic considerations, a growers' yield is best considered as the output harvest for cultivation area (g/m^2^), rather than for a single plant (g/plant). In all plant architecture treatments evaluated in this study, inflorescence biomass yield production per m^2^ was higher in the higher density treatment compared with the lower density treatment, except for the “Pruning” treatment that was not significantly affected by plant density (Table 1). In many crop species, changing plant density was reported to affect yield biomass/m^2^ as well as yield quality (Islam et al., 2011; Maboko et al., 2011; Hozayn et al., 2013). Increased density was found to increase yield (Hozayn et al., 2013) but also to reduce yield quality (Maboko et al., 2011), suggesting the existence of an optimum density that needs to be determined for each production goal. As cannabis is prized for its chemical components, it could be compared to aromatic herbs, as their value is defined mostly by the secondary metabolites rather than solely by yield biomass. Similar to our results for cannabis, in basil (*Ocimum basilicum*), parsley (*Petroselinum crispum*) and chamomile (*Matricaria chamomilla*), leaves and floral yield increased with the increase in plant density (Pirzad et al., 2011; Bekhradi et al., 2014; El-Zaeddi et al., 2016) but not in dill (*Anethum graveolens*) (Callan et al., 2007). The interplay between plant density and architectural manipulation was seen in tomatoes where total yield increased with increased density and with reduced stem pruning (Maboko et al., 2011).

Several studies involving planting density were conducted on hemp-type cannabis in the past, but the yield tested in those studies was biomass for animal feed, fibers, or seeds (Amaducci et al., 2002; Grabowska and Koziara, 2006), under cultivation practices that vary considerably from drug-type cannabis agrotechniques. A study by Campiglia et al. (2017) did however test inflorescence yield and found that higher plant density resulted in improved floral yield in seven genotypes, but the effect on inflorescence chemical composition was not tested. A meta-analysis of *Cannabis sativa* yield for data reported by previous studies, point at the use of low plant density, ≤ 12 plants per square meter, for increased cannabis yield per square meter (Backer et al., 2019).

In the present study, cannabinoid concentrations in the plant, calculated as the plant average concentration (Supplementary Figure 2) were mostly reduced (by up to 24%) or not affected by the increase in density (excluding “BBLR” CBDA and CBDVA, which were increased by up to 18%). However, the increase in inflorescence yield biomass per cultivation area under dense plant cultivation compensated for the reduced concentrations, and the cannabinoid yield per cultivation area (mg/m^2^) (Table 2) was, therefore, not affected. Effect of plant density on secondary metabolites production per cultivation area may vary between crops; in lavender (*Lavandula hybrida*), decreased intra-plant spacing increased secondary metabolite production (Arabaci et al., 2007).

Cannabis inflorescence is a rather unique plant-remedy since it is used today by western medicine mainly as an intact plant material or its extract. While other plant-based medicinal compounds are extracted from plants (if synthetic production is not possible or is less economical) and are dosed at a well-defined concentration into modern drugs with known specific effects. As such, it is still not fully known how varying amounts and ratios of the cannabis components affect the treatments' efficacy. In this study, a larger variability in concentrations and ratios between identified cannabinoids was identified in the densely grown plants compared with the more spaced stand. In the treatments of this density, 9–19% of the inflorescences in a plant varied in concentration by more than 50% from the average plant concentration (Table 3). This alarmingly large variation might impose a problem for inflorescence-based therapy, but less so for mass production of extracts which allows standardization of some of the compounds. According to a previous study (Danziger and Bernstein, 2021b), with smaller plants cultivated at a similar (1 plant/m^2^) density, the chemical variation in the smaller plants was generally lower. Until further studies evaluating the effects of varying inflorescence chemical profile on cannabis-treated patients will be conducted, it will not be possible to determine whether the added value of increased yield outweighs the reduced uniformity under higher density.

### Light Effects

Light is a key factor effecting plant growth and development (Kami et al., 2010). Higher light irradiance is connected to faster growth and higher yields (Eaves et al., 2020). However, even under high irradiance, some high-intensity crops suffer from insufficient light levels when dense canopy prevents sufficient light from reaching lower parts of the plant (Fowler and Reta-sanchez, 2002). Under higher density growth, cannabis inflorescences showed decreased cannabinoids synthesis at the lower parts of the plants, leading to an increase in spatial chemical variability (Figure 5). This decrease could be attributed to lower light penetration through the denser canopy (Supplementary Figure 3). In numerous crops, including cotton, light penetration to the canopy was found to have a profound effect on yield (Chapepa et al., 2020), and a similar reduction in yield was observed in this study (Figure 2B). The chronic lack of light at the bottom of the highly dense plants is suggested also by the increased degradation of leaves and branches in the high-density treatments (Figure 1), as was formerly described for a range of plants including *Arabidopsis thaliana* (Weaver and Amasino, 2001). Such degradation is reported to be highly localized, which explain why only the bottom-most branches senesced, while branches from higher and more external locations showed little variance. The earlier senescence of the lower leaves and branches corresponds also with the localized effect on the chemical profile of inflorescence from the lower parts of the plant (Figure 5).

Plants have developed various mechanisms to cope with the reduction of light penetration through the canopy. According to Slattery et al. (2017), reduced leaf chlorophyll contents lowers photosynthetic rate at the upper leaves but increases light penetrance to the canopy without reducing yield. Our results reveal a trend for similar adaptation mechanism in cannabis, with significant reductions in most pigments in the BBLR and pruning treatments in the more densely grown plants (and a similar albeit not significant trend in the control treatment) (Figure 7). A similar trend was reported also for pepper and basil, as high-density planting reduced leaf chlorophyll contents (Aminifard et al., 2010; Abdou et al., 2017). Since the leaves analyzed for pigment quantification in our study were located at the upper third of the canopy, shading could not have influenced pigment biosynthesis. This suggests that other elicitors induced these observed changes in chlorophyll accumulation. Possible effectors are hormonal changes such as gibberellin, which stimulates plant elongation under low light but whose presence is also associated with lower chlorophyll content per area (Liu et al., 2017; Yan et al., 2020). This reasoning is strengthened by the elongated plants that developed under the high-density cultivation, as was previously seen in many crops such as tomatoes (Ohta et al., 2018) and corn (*Zea mays*) (Maddonni et al., 2001). Results for membrane leakage and osmotic potential in leaves from the upper 3rd of the plants reflect as well impact of planting density on the physiological status of the plants. Membrane leakage and osmotic potential were generally higher and lower, respectively, in upper leaves of the higher density plants, presenting a negative effect of high density on the physiological state (Figure 7). It should be noted that very high plant densities (>20 plants/m^2^) reduced cannabis hemp-type plant height as was described previously (Amaducci et al., 2002; Bhattarai and Midmore, 2014). However, since industrial fiber-hemp morphology differs from drug-type medical cannabis, and the growth patterns were bred for different production goals, developmental and physiological responses are expected to differ. It is, therefore, not possible to predict responses of medical cannabis plants from industrial fiber-hemp results.

In our study, two treatments changed light penetration to the canopy, “Defoliation,” by removing most of the leaves obscuring the light, and “BBLR” that involved removal of all leaves, branches, and inflorescences from the bottom of the plant thus eliminating tissue that receive insufficient light levels (Supplementary Figure 3). Light penetrance to the canopy was substantially higher in the “Defoliation” treatments compared to all other treatments, though this change was made late in development and its effect was, therefore, limited. This is unlike the “BBLR” treatment, which eliminated growth in the shaded lower parts of the plant altogether throughout the growing season. Defoliating the plants resulted in reduced yield biomass per plant under the high density, but increased cannabinoid concentrations at the bottom of the plants compared to the “Control.” It is possible that the yield reduction results from inhibition of inflorescence growth by a chronic lack of light throughout the season prior to the defoliation, similar to the effect on the “control,” and that the loss of leaves that was imposed at the end of the season occurred too late in development to compensate for the reduction in inflorescence yield by the chronic lack of light. However, cannabinoid synthesis was improved by the added light at the end of the season following defoliation, during chemical maturation. Unlike the “Defoliation” treatment, “BBLR” has not caused a decrease in yield compared to the control under both density treatments, which could be explained by the timing of the treatment that was imposed on the plants early at the flowering stage thus preventing inhibition of floral development due to lack of light.

To mitigate yield loss and reduction of secondary metabolites, it is possible to introduce artificial light into the canopy. The use of intra-canopy lights is becoming more prevalent to increase yield at the lower sun-deprived parts of plants (Davis and Burns, 2016). In cannabis, a single study used sub-canopy LED lights that were shown to increase yield quantity as well as the cannabinoid contents at the bottom third of the plant (Hawley et al., 2018). In addition, several studies evaluated different spectral properties on cannabis development, yield and its components showing differential response to light quality (Magagnini et al., 2018; Eaves et al., 2020; Bevan et al., 2021; Danziger and Bernstein, 2021a) as well light intensity (Potter and Duncombe, 2012). As light travels through the plant canopy, different wavelengths are absorbed by the plant organs altering its spectrum as well as intensity (Kasperbauer, 1971). We therefore suggest that a combined approach of spectral quality optimization inside the canopy, and increased light intensity by architectural modulation treatments or sub-lighting illumination can be utilized to improve yield components in cannabis grown in high density.

## Conclusions

In this study, we evaluated the effects of plant architecture and plant density on growth, development and chemical properties of medical drug-type) *Cannabis sativa* plants. We tested the hypotheses that an increase in plant density will increase inflorescence yield per area while reducing chemical quality and uniformity, and furthermore, that manipulating plant architecture will interact with such variations. The results indicated that an increase in plant density decreased inflorescence yield per plant but increased yield per area (except in the pruning treatment that was not affected significantly) thus supporting the hypotheses. In addition, cannabinoid concentrations were reduced in the lower part of the plant by the increase in plant density (except in the BBLR treatment that did not have true lower branches), but were generally unchanged (or much less affected) in the top apical inflorescence- thus highly reducing the cannabinoid uniformity across the plant. Plant biomass was reduced by the higher plant density, while plant height was increased. The information gained in this study can direct cannabis growers to customize cultivation practices to target the final product goals, in terms of yield quantity vs. chemical quality.

## Data Availability Statement

The original contributions presented in the study are included in the article/Supplementary material, further inquiries can be directed to the corresponding authors.

## Author Contributions

NB conceptualization, Funding acquisition, supervision, and planning the experiments. ND carried out the experiments. NB and ND wrote the manuscript. Both authors contributed to the article and approved the submitted version.

## Funding

This work was funded by the Chief scientist fund of the Israeli Ministry of Agriculture, Israel, Grant No. 20-03-0049.

## Conflict of Interest

The authors declare that the research was conducted in the absence of any commercial or financial relationships that could be construed as a potential conflict of interest.

## Publisher's Note

All claims expressed in this article are solely those of the authors and do not necessarily represent those of their affiliated organizations, or those of the publisher, the editors and the reviewers. Any product that may be evaluated in this article, or claim that may be made by its manufacturer, is not guaranteed or endorsed by the publisher.
