# Supplementary material for: Too Dense or Not Too Dense: Higher Planting Density Reduces Cannabinoid Uniformity but Increases Yield/Area in Drug-Type Medical Cannabis
Source: Front Plant Sci. 2022 Sep 29;13:713481. doi: 10.3389/fpls.2022.713481 (PMC9559401; doi:10.3389/fpls.2022.713481)
Supplement: Supplementary file 1 [file Data_Sheet_1.pdf]

Supplemental material

Too dense or not too dense: Higher planting density reduces cannabinoid uniformity but increases yield per area in drug-type medical cannabis

Nadav Danziger, Nirit Bernstein*

Institute of Soil water and Environmental Sciences, Volcani Center, Israel, Nirit@agri.gov.il

* Corresponding author: E-mail address: Nirit@agri.gov.il ; Tel: 972 506 220707

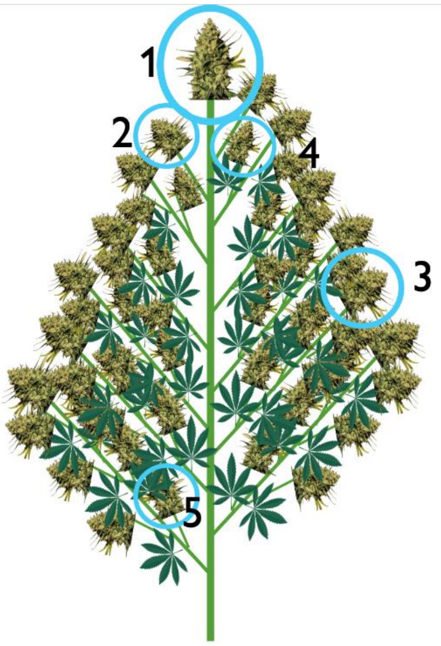

Supplemental Fig. 1: The five locations in the plant sampled for cannabinoid concentrations:

1. The top most inflorescence;
2. The apical inflorescence of a high branch (the 4th highest branch);
3. Apical inflorescence of a low branch (4th from plant base);
4. An axillary inflorescence located close to the stem at the top area of the plant (2nd branch from the top);
5. The bottom most inflorescence located closest to the stem (an axillary inflorescence from the lowest branch closest to the stem).

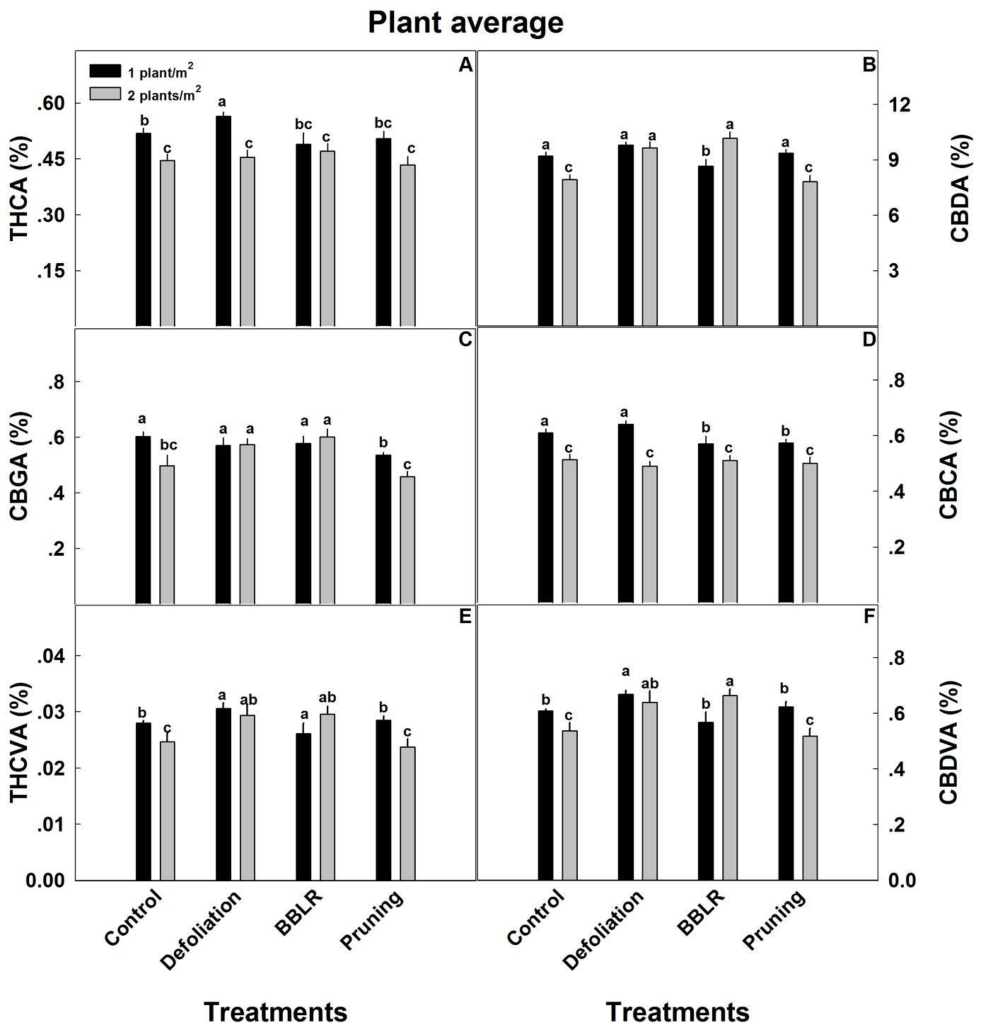

Supplemental Fig. 2. 'Plant-average' Cannabinoid concentrations in cannabis plants grown under two densities (1 or 2 plants m^{-2}) and four architectural modulation treatments. THCA (A), CBDA (B), CBGA (C), CBCA (D), THCVA (E) and CBDVA (F). The results are mean and SE (n=6). Different letters above the bars represent significant differences between treatments by Tukey HSD test at α=0.05.

'BBLB' = removal of leaves and branches from the bottom part of the plant.

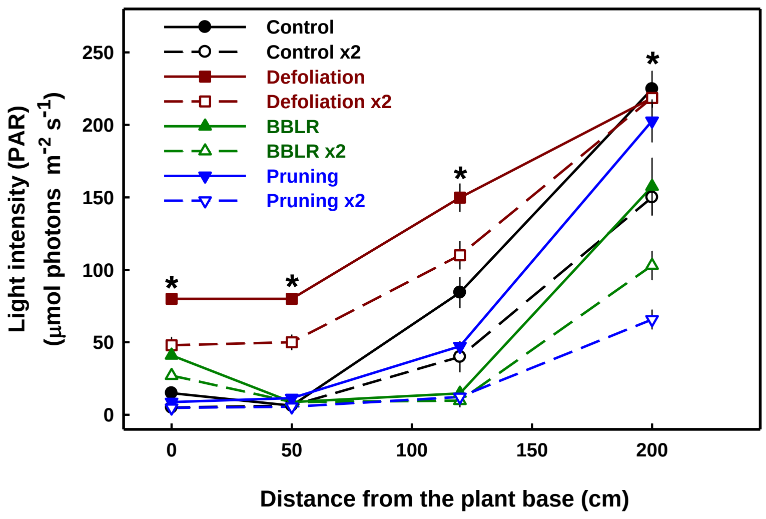

<u>Supplemental Fig. 3.</u> Effect of the treatments on light intensity at different heights along the plants. Light level at the greenhouse above the canopy was 320 (µmol photons m^{-2} s^{-1}). Data are averages ± SE (n = 6). Asterisks above the averages represent significant differences between treatments at a given longitudinal location, by the Tukey HSD test at α = 0.05.
